# Cold Shock Domain Protein DbpA Orchestrates Tubular Cell Damage and Interstitial Fibrosis in Inflammatory Kidney Disease

**DOI:** 10.3390/cells12101426

**Published:** 2023-05-19

**Authors:** Jonathan A. Lindquist, Anja Bernhardt, Charlotte Reichardt, Eva Sauter, Sabine Brandt, Rajiv Rana, Maja T. Lindenmeyer, Lars Philipsen, Berend Isermann, Cheng Zhu, Peter R. Mertens

**Affiliations:** 1 Department of Nephrology and Hypertension, Diabetes and Endocrinology, Otto-von-Guericke-University Magdeburg, 39120 Magdeburg, Germany; jon.lindquist@med.ovgu.de (J.A.L.); anja.bernhardt@med.ovgu.de (A.B.); charlotte.reichardt@med.ovgu.de (C.R.); eva.wollmesheim@gmail.com (E.S.); sabine.brandt@med.ovgu.de (S.B.); zhuc@srrsh.com (C.Z.); 2 Institute of Laboratory Medicine, Clinical Chemistry and Molecular Diagnostics, University Hospital Leipzig, Leipzig University, 04103 Leipzig, Germany; rajiv.rana@medizin.uni-leipzig.de (R.R.); berend.isermann@medizin.uni-leipzig.de (B.I.); 3 Department of Medicine, University Medical Center Hamburg-Eppendorf, 20251 Hamburg, Germany; m.lindenmeyer@uke.de; 4 Hamburg Center for Kidney Health (HCKH), University Medical Center Hamburg-Eppendorf, 20251 Hamburg, Germany; 5 Institute of Molecular and Clinical Immunology, Otto-von-Guericke-University, 39120 Magdeburg, Germany; lars.philipsen@med.ovgu.de; 6 Department of Nephrology, Sir Run Run Shaw Hospital, Zhejiang University, School of Medicine, Hangzhou 310058, China

**Keywords:** kidney injury, kidney fibrosis, cold shock proteins

## Abstract

DNA-binding protein A (DbpA) belongs to the Y-box family of cold shock domain proteins that exert transcriptional and translational activities in the cell via their ability to bind and regulate mRNA. To investigate the role of DbpA in kidney disease, we utilized the murine unilateral ureter obstruction (UUO) model, which recapitulates many features of obstructive nephropathy seen in humans. We observed that DbpA protein expression is induced within the renal interstitium following disease induction. Compared with wild-type animals, obstructed kidneys from *Ybx3*-deficient mice are protected from tissue injury, with a significant reduction in the number of infiltrating immune cells as well as in extracellular matrix deposition. RNAseq data from UUO kidneys show that *Ybx3* is expressed by activated fibroblasts, which reside within the renal interstitium. Our data support a role for DbpA in orchestrating renal fibrosis and suggest that strategies targeting DbpA may be a therapeutic option to slow disease progression.

## 1. Introduction

Renal fibrosis resulting from excessive deposition of the extracellular matrix is a common feature of chronic kidney disease (CKD) [1,2]. Fibrotic deposits alter the tissue architecture, resulting in decreased renal function that ultimately leads to organ failure. Currently, there is limited therapy that can slow or stop disease progression [3]. Therefore, a better understanding of the underlying molecular mechanisms that contribute to fibrogenesis may identify new targets for therapeutic intervention.

Y-box binding protein-1 (YB-1; gene *Ybx1*) and DNA-binding protein A (DbpA; gene *Ybx3*) belong to the Y-box family of cold shock domain proteins that bind single-stranded nucleic acids via their evolutionarily conserved cold shock domain [4,5]. Therefore, these proteins play a central role in regulating a number of cellular processes, including transcription, translation, and the cell cycle. We have shown that kidney injury induces the expression of both YB-1 and DbpA [6,7]. While YB-1 is a key regulator of fibrosis-related genes, less is known about the function(s) of DbpA [4,8,9].

DbpA was first identified as a transcription factor that binds to a responsive element within the epidermal growth factor receptor (EGFR) promoter and is known by several names, including mouse Y-box protein 3 (MSY3), mouse Y-box protein 4 (MSY4), cold shock domain A (CSDA), and zonula occludens-1 (ZO-1)-associated nucleic acid binding protein (ZONAB) [10]. There are two isoforms of DbpA that result from alternative splicing, hence the names MSY3 and MSY4 [11]. Both isoforms possess an ancestral cold shock domain, but differ by a single exon, encoding 69 amino acids. DbpA is mainly expressed in smooth muscle cells of the heart, vasculature, and skeletal muscle, whereas YB-1 is ubiquitously expressed [12,13]. Upregulated DbpA expression has been observed in various cancers [14,15,16,17,18,19]. DbpA was first described as a component of tight junctions, due to its interaction with ZO-1 (also known as tight junction protein 1). When cells lose their cell–cell contacts, DbpA is released from the plasma membrane and translocates to the nucleus, where it triggers cell proliferation by regulating target genes such as thymidine kinase, cyclin D1, and proliferating cell nuclear antigen (PCNA) [20,21,22,23,24,25].

DbpA shows a strong expression in developing embryos, but is rapidly downregulated after birth [21]. Congruently, we recently demonstrated that the DbpA protein was barely detected in healthy kidney tissue; however, its expression was strongly induced upon the induction of mesangioproliferative glomerulonephritis [7]. DbpA also plays a crucial role in the differentiation and proliferation of proximal tubular cells [21]. However, little is known about the role of DbpA in kidney disease. The aim of this study was to determine the contribution of DbpA to disease progression. Therefore, we applied a surgical model of ureteral obstruction to wild-type and *Ybx3*-deficient mice. This model recapitulates many features of obstructive nephropathy in humans, including subacute renal injury characterized by tubular cell damage, interstitial inflammation, and the development of fibrosis [26,27].

## 2. Materials and Methods

### 2.1. Animals

Animal studies were performed using genetically modified mice on a C57BL/6N background. The animals were housed and bred in the Central Animal Facility of the Medical Faculty at the Otto-von-Guericke University Magdeburg as previously described [28].

### 2.2. Genotyping Analyses

First, the tissue biopsy was lysed with 200 µL direct PCR lysis buffer and 50 µg of proteinase K. PCR was performed with primer pairs for mouse MSY4 (MSY4F3: CAGACTCAGAAAATGAAGGGCA; MSY4R3: AGATGGGCAAGATTCTCAAGTC; and PGK1: TGAGACGTGCTACTTCCATTTG) under the following conditions: MSY4 PCR, 35 cycles at 95 °C for 30 s, 58 °C for 30 s, and 72 °C for 60 s. Products were separated on 2% agarose gels.

### 2.3. Animal Experiments

Unilateral ureter obstruction was performed on 14 healthy C57BL/6N mice (Janvier, 20 to 30 g, 12 to 14 weeks of age) and 13 healthy MSY4-/- mice (T. Ley, Washington University School of Medicine, St. Louis, MO, USA) as previously described [28].

### 2.4. Isolation of Kidney Cells

The kidney tissue was mechanically crushed, resuspended in digestion buffer (RPMI, 0.1% BSA, 1 mg/mL collagenase D, and 100 µg/mL DNase I), and incubated for 30 min (37 °C, 5% CO_2_, 100% humidity). After repeated mechanical grinding, the entire contents of the well were placed on a 70 µm cell strainer, rinsed, and then placed and rinsed on a 40 µm cell strainer. After centrifugation, erythrocytes were lysed, and the cells were washed and analyzed via flow cytometry [29].

### 2.5. Flow Cytometry

Cells (1 × 10^6^) were used for the staining procedure. The cells were stained with a directly labeled antibody for 30 min at 4 °C in the dark. Fixing and permeabilization of the cells were performed using the Fix/Perm buffer set (BioLegend, San Diego, CA, USA) according to the manufacturer’s instructions. To prevent non-specific binding, the cells were incubated with 5% mouse serum for 10 min at 4 °C. The list of surface markers includes: CD11b clone M1/70 APC/Cy7, CD3 clone 145-2C11 APC, CD45 clone 30-F11 PE, CD73 clone TY/11.8 Alexa Fluor^®^ 647 (all from BioLegend), and PDGFRβ clone APB5 PE (AbCam, Cambridge, UK). The flow cytometry measurements were performed using a FACSAria II instrument (BD Immunocytometry Systems). Data collection and analysis were performed using the FlowJo software version 10. Controls were used for gating analyses to distinguish positively from negatively stained cell populations (Supplementary Figure S1).

### 2.6. TaqMan Real-Time PCR Assay

The total RNA of the kidney was extracted using an RNAeasy mini kit (Qiagen Inc., Valencia, CA, USA) according to the manufacturer’s instructions. The total RNA (1 μg) was reverse-transcribed using a SuperScript III First-Strand Synthesis System (Thermo Fischer Scientific Inc., Rockford, IL, USA). The real-time PCR reactions were performed on a 7500 Fast Real-Time PCR System (Applied Biosystems, Foster City, CA, USA) using TaqMan Universal PCR Master Mix (Applied Biosystems). Collagen type I, collagen type III, and 18S rRNA mRNAs were detected using FAM-labeled specific primers purchased from Applied Biosystems. The thermal cycling conditions were as follows. After an initial hold of 2 min at 50 °C and 10 min at 95 °C, the samples were cycled 40 times at 95 °C for 15 sec and 60 °C for 1 min. Relative levels of αSMA, collagen type I, and collagen type III mRNAs in each sample were evaluated after normalization with 18S expression. Beta-Actin (Actb) (Mm00607939_s1) served as an internal control (Life Technologies GmbH, Darmstadt, Germany). The relative abundance (fold change against uninfected controls) was calculated using the delta Ct method (2^−ΔΔCt^).

### 2.7. Immunohistochemistry

Methacarn-fixed, paraffin-embedded tissue sections were used for immunohistochemistry and immunofluorescence staining as previously described [28].

### 2.8. Periodic Acid–Schiff (PAS) Staining

The staining procedure was performed as previously described [28].

### 2.9. Sirius Red Staining

Kidney fibrosis was quantified using Sirius red staining as previously described [28].

### 2.10. Western Blot Analysis

Tissue protein extracts were prepared and separated via SDS-PAGE (10% Bis-Tris gels) and transferred onto nitrocellulose membranes (Bio-Rad Laboratories, Inc., Hercules, CA, USA). Non-specific binding was blocked with 5% milk in TBS-tween, before adding primary antibodies: anti-YB-1 C-terminal (aa 306-321, Eurogentec, Seraing, Belgium), anti-DbpA N-terminal (aa 51-63, Eurogentec), anti-GAPDH (14C10, Cell Signaling, Danvers, MA, USA), anti-PDGFRβ (28E1, Cell Signaling), anti-β-catenin (Cell Signaling), anti-vinculin (V284, Santa Cruz, CA, USA), anti-E-cadherin (24E10, Cell Signaling), anti-MSY2 (ab154829, Abcam). Horseradish peroxidase-conjugated anti-rabbit or anti-mouse secondary antibodies (Southern Biotech, Birmingham, AL, USA) were used for detection. Antibodies were visualized using SuperSignal chemiluminescence substrate Pierce ECL (Thermo Fischer Scientific) according to the manufacturer’s instructions.

### 2.11. Automated Multidimension Microscopy

Automated multidimension microscopy was performed as previously reported [28].

### 2.12. Human Microarray Analysis

Human renal biopsy specimens were collected in an international multicenter study, the European Renal cDNA Bank-Kröner–Fresenius Biopsy Bank (ERCB-KFB) [30]. Biopsies were obtained from patients after receiving informed written consent and with approval of the local ethics committees. Biopsies were prepared as previously reported [31]. Glomerular and tubular samples were analyzed for mRNA expression levels (GSE32591, GSE35489, GSE37463, GSE47185, GSE 99340). CEL file normalization was performed with the Robust Multichip Average method using RMAExpress (Version 1.20) and the human Entrez-Gene custom CDF annotation from Brain Array version 25 (http://brainarray.mbni.med.umich.edu/Brainarray/Database/CustomCDF/CDF_download.asp, accessed on 28 November 2022). The log-transformed dataset was corrected for a batch effect using ComBat from the GenePattern pipeline (http://www.broadinstitute.org/cancer/software/genepattern/, accessed on 28 November 2022). To identify differentially expressed genes, the SAM (significance analysis of microarrays) method was applied using the SAM function in Multiple Experiment Viewer (TiGR MeV, Version 4.9) [32]. A q-value below 5% was considered to be statistically significant.

### 2.13. Statistical Analyses

The results were calculated and presented as means ± SDs. The Student *t*-test was applied for two-group comparisons, with * *p* < 0.05 considered statistically significant; ** *p* < 0.01, *** *p* < 0.001.

## 3. Results

### 3.1. Verification and Characterization of the Ybx3 Knockout

**Figure 1:**
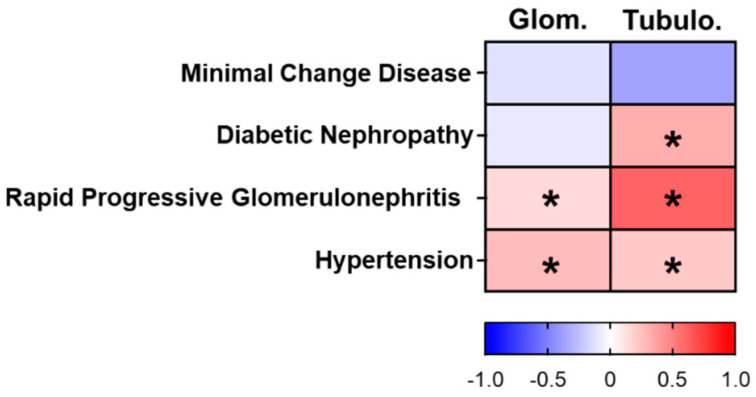
Summary of the *YBX3* gene expression data of microdissected glomerular (Glom.) and tubulointerstitial (Tubulo.) compartments from patients with different kidney diseases. Data are presented as a heatmap of the log fold change. Red-marked genes mean upregulated and blue-marked genes mean downregulated in comparison to the control (living donor). Asterisks indicate a q-value < 5% and are therefore to be regarded as significantly regulated. Diabetic nephropathy (DN; Glom: n = 14, Tub: n = 18); hypertensive nephropathy (HTN; Glom: n = 15, Tub: n = 21); minimal change disease (MCD; Glom: n = 14, Tub: n = 15); rapidly progressive glomerulonephritis (RPGN; Glom: n = 23, Tub: n = 21); controls (living donors (LD); Glom: n = 41. Tub: n = 42).

We first analyzed the changes in *YBX3* expression in both the glomerular and tubulointerstitial compartments of microdissected patient biopsies. Our data suggest disease-specific changes in *YBX3* transcript levels (Figure 1). To further investigate the role of DbpA in kidney disease progression, we examined a model of tubulointerstitial nephritis, namely unilateral ureter obstruction (UUO), in wild-type and MSY4-deficient mice, hereafter referred to as *Ybx3*-deficient mice.

**Figure 2:**
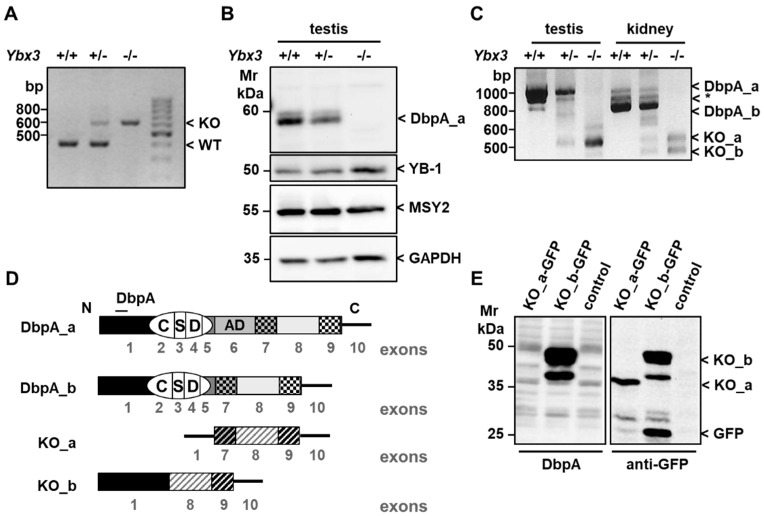
Deletion of exons 2–5 within the *Ybx3* gene leads to the formation of an alternative translation/splice product. (**A**) Genotyping of tail biopsies showed the presence of the expected PCR products at ~400 bp and ~600 bp for wild-type and knockout mice, respectively. (**B**) DbpA protein expression was assessed via Western blotting of testis tissue using an anti-MSY4 antibody recognizing the C-terminal domain of DbpA (residues 249 to 263) [33]. (**C**) PCR amplification of the whole *Ybx3* coding sequence detected modified mRNA products from the knockout mice (*KO_a* and *KO_b*). Sequence analyses identified two protein products in knockout mice. One was previously reported as *CSDA CRA_a* (denoted KO_a), and the second is a fusion of the *Ybx3* N-term (exon 1) with parts of CSDA CRA_a (denoted KO_b). (**D**) The cartoon depicts the relationship between the exons and coding sequences (filled boxes). The cold shock domain (CSD) is indicated as well as the N- and C-terminus. The DbpA isoforms differ by the presence or absence of the alternative domain (AD) encoded in exon 6. The 3′ untranslated region (UTR) encoded in exon 10 is indicated by a line. Alternatively translated exons are indicated with stripped bars. (**E**) The *KO-a* and *KO-b* fragments were subcloned into the pEGFP vector and transiently expressed in HEK293 cells. Western blot analysis detected both KO_a (37 kDa) and KO_b (40 kDa and 48 kDa) proteins in transfected HEK293 cells. KO_b could be detected with an anti-DbpA antibody directed against epitopes within the protein N-terminus. The lower MW band may result from alternative splicing.

As previously reported, deletion of the *Ybx3* gene has no overt effect on either the birth rate or development of knockout animals; however, male mice display decreased fertility after 4 months of age [33]. Deletion of the gene was confirmed by both PCR genotyping (Figure 2A) and Western blotting (Figure 2B). Since DbpA is expressed in two isoforms, we isolated mRNA from the testis and kidney of wild-type, heterozygous, and knockout mice to determine which isoform(s) is (are) expressed in the kidney [11]. Primers were designed corresponding to the N- and C-terminus of the DbpA coding sequence, which should yield products of 1083 and 875 bp, respectively. Analysis of the amplification products identified three bands present in both the testis and kidney of wild-type mice, which were reduced in intensity in the heterozygote, and absent in the knockout (Figure 2C). Sequence analysis confirmed the identity of the upper and lower bands as the long and short isoforms of DbpA, i.e., DbpA_a and DbpA_b, respectively. The identity of the third band (*), provided by the Proteomics Database, may be a premature termination product of the long isoform reported in humans [34]. Thus, our analysis showed that the long isoform DbpA_a was predominantly expressed in the testis, which is consistent with the Western blot results, whereas DbpA_b predominated in kidney tissue. Generation of the knockout induced truncated RNA products, which were not seen in wild-type tissue. Sequencing confirmed that the upper band, indicated as KO_a, corresponds to the expected truncated mRNA product containing exons 1, 7, 8, and 9 of the *Ybx3* gene (Figure 2D) [33]. This truncated message encodes a second open reading frame that corresponds to the 113-amino-acid sequence of the cold shock domain protein A, isoform CRA_a (Supplementary Figure S2A) [35]. Further analysis showed that the lower band visible in the knockout kidney, indicated as KO_b, results from alternative splicing and lacks exon 7. This mRNA encodes a potential fusion protein consisting of the 79 N-terminal residues of DbpA fused to the last 86 residues of CRA_a (Supplementary Figure S2B). To test whether this mRNA encodes a functional protein, we cloned the PCR products into the vector pEGFP-N1. Western blot analysis of transfected cell lysates with N-terminal anti-DbpA sera (aa 51-63) and anti-GFP confirmed the existence of CRA_a as well as the fusion protein, with products visible in the 35–50 kDa range (Figure 2E). This is the first report that shows CSDA_CRA_a encodes a gene product of unknown function.

**Figure 3:**
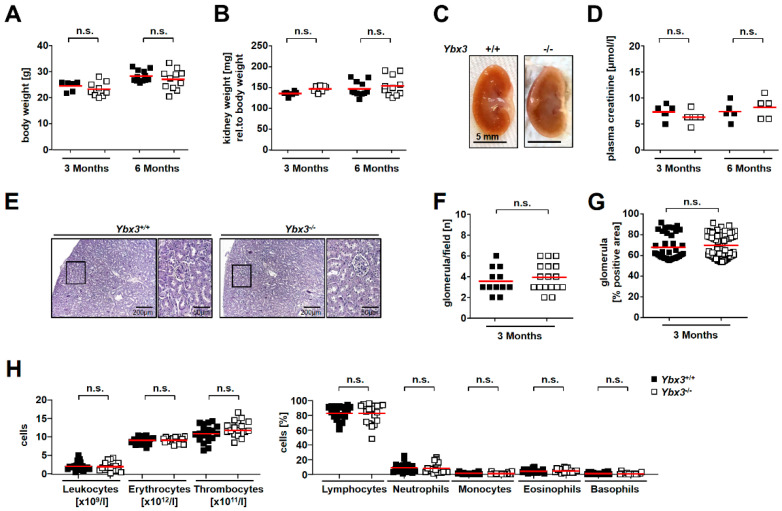
*Ybx3* knockout mice develop normally. (**A**) The body weight of wild-type (black) and *Ybx3*-deficient mice (white) was comparable at 3 and 6 months of age. (**B**) The weight of the kidneys at 3 and 6 months of age is shown after normalization to the body weight. (**C**) Photographs of whole kidneys from wild-type and *Ybx3*-deficient animals showed no morphological differences. (**D**) Kidney function (i.e., plasma creatinine levels) was similar in wild-type and knockout animals. (**E**) Periodic acid–Schiff (PAS) staining of kidney tissue slices revealed no structural differences between wild-type and *Ybx3* knockout mice; scale bars represent 200 µm and 50 µm. The number (**F**) and size (**G**) of the glomeruli per visual field were comparable between wild-type and knockout animals. (**H**) Blood counts revealed no alterations in the cell number between wild-type and knockout animals.

The growth of the knockout mice was comparable with that of their wild-type littermates, despite a trend of reduced weight. *Ybx3*-deficient mice showed normal kidney development and function, and a normal distribution of blood cells, indicating that hematopoiesis is not impaired (Figure 3A–H).

**Figure 4:**
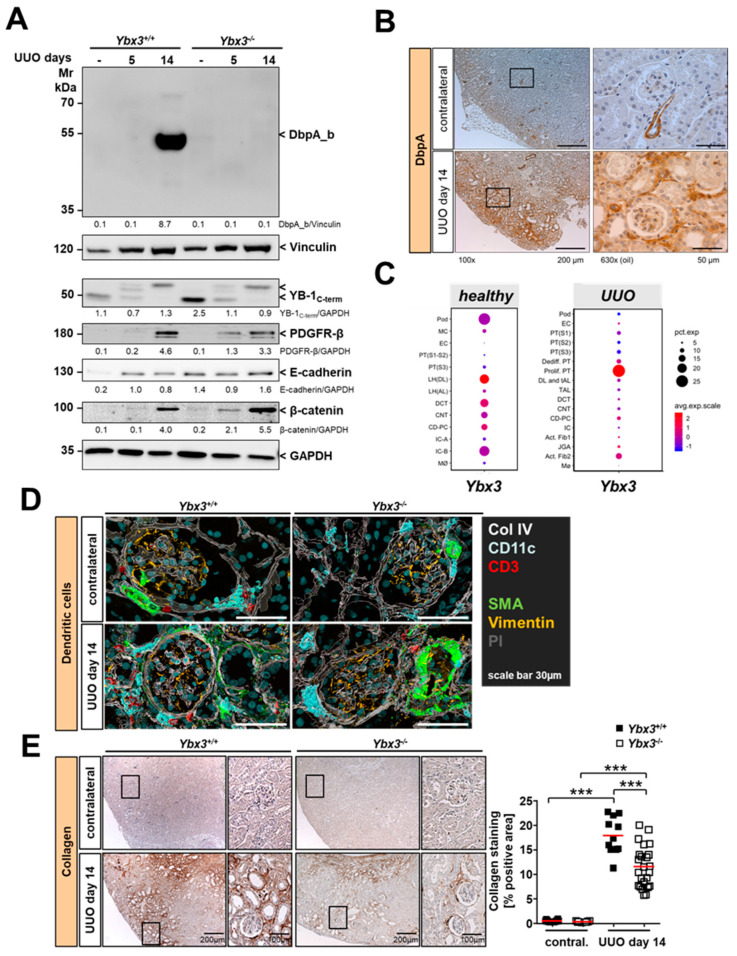
The DbpA protein is induced following ureter ligation. To investigate the role of DbpA in tubulointerstitial nephritis, we utilized the murine unilateral ureteral obstruction (UUO) model. (**A**) Western blot analysis of contralateral or ligated kidney lysates revealed massively upregulated DbpA expression following UUO in wild-type animals on day 14. PDGFR-β, E-cadherin, and β-catenin served as markers for successful disease induction, while GAPDH served as a loading control. Apparent molecular weights are indicated in kDa. (**B**) Immunohistochemistry using the N-term DbpA antibody showed profound induction of DbpA protein expression in the interstitium of the kidney in UUO compared to the unaffected contralateral kidney tissue. Staining of the blood vessels served as a positive control in healthy kidneys. Scale bars correspond to 200 μm and 50 μm as indicated. (**C**) Single-cell RNA transcriptomics data were analyzed using the KIT software (http://humphreyslab.com/SingleCell/, accessed on 22 April 2022) [42,43]. Expression data for *Ybx3* in healthy mouse kidneys before and after 14 days of UUO are shown. Abbreviations: Pod—podicyte; MC—mesangial cell; EC—endothelial cell; PT—proximal tubule; LH(DL)—loop of Henle descending loop; LH(AL)—loop of Henle ascending loop; DCT—distal convoluted tubule; CNT—connecting tubule; CD-PC—collecting duct-principal cell; IC-A—intercalated cell type A; IC-B—intercalated cell type B; MØ—macrophage; Dediff. PT—dedifferentiated PT; Prolif. PT—proliferating PT; Act. Fib.—activated fibroblast; JGA—juxtaglomerular apparatus; DL+tAL—descending loop + thin ascending loop. (**D**) Automated multidimensional fluorescence microscopy of diseased (UUO) kidney cryosections from wild-type and *Ybx3*-deficient mice. The architecture of the kidney was visualized using collagen IV staining (white) to show the extracellular matrix; vimentin staining (yellow) depicts the glomeruli, and propidium iodide (PI, gray) staining depicts the nuclei. Tissue-resident dendritic cells were visualized using CD11c (cyan) and infiltrating T cells CD3e (red). A scale bar of 30 μm is indicated. (**E**) Immunohistochemistry of collagen type I served as a marker of renal fibrosis. Scale bars corresponding to 200 μm and 100 μm are indicated. Quantification is provided for wild-type and knockout animals (*** *p* < 0.001).

Consistent with published results showing that *Ybx3* mRNA is downregulated during kidney development, we previously reported that the DbpA protein is not detectable in healthy kidney tissue; however, DbpA expression is induced in mesangioproliferative diseases [7]. Therefore, we first analyzed tissue lysates 5 and 14 days after UUO induction to determine to what extent DbpA was expressed (Figure 4A). Immunostaining with anti-DbpA serum showed a strong induction of a 50 kDa protein corresponding to the short isoform DbpA_b at 14 days in kidney lysates from wild-type mice, which was not observed in the knockout. The induction of platelet-derived growth factor receptor-β (PDGFR-β), E-cadherin, and β-catenin served as a control for successful disease induction, whereas glyceraldehyde-3-phosphate dehydrogenase (GAPDH) staining showed that comparable amounts of tissue lysates were loaded [36,37,38]. Activation of YB-1 was also seen upon disease induction, as previously reported [28]. E-cadherin and YB-1 expression appeared enhanced in the *Ybx3* knockout. The latter finding is supported by observations that these cold shock proteins counter-regulate one another [39,40,41]. Immunohistochemistry showed that in healthy kidneys, DbpA was primarily expressed only in vascular smooth muscle cells, which is in agreement with our previous observation [7]. However, after UUO induction, a dramatic upregulation of DbpA expression was observed within the kidney cortex (Figure 4B). Closer examination showed that DbpA was primarily expressed in the interstitium, with little or no staining visible in the glomeruli or tubuli. DbpA showed a membranous staining in the vasculature of healthy kidneys, which fits the reported junction-associated activities of DbpA [11,21]. In contrast, the disease-induced DbpA showed greater nuclear staining after UUO induction. Published single-cell RNA sequencing data from kidneys of UUO mice show an induction of *Ybx3* expression in proliferating proximal tubular cells and activated fibroblasts (Figure 4C) [42,43]. Surprisingly, the pattern of DbpA expression in the kidney resembles that of tissue-resident dendritic cells (Figure 4D) as well as type I collagen (*Col1a1*), which is upregulated in the tubulointerstitium during fibrosis (Figure 4E) [2,44,45,46]. Both collagen I staining and the number of CD11c-positive cells were reduced in obstructed *Ybx3*-deficient kidneys compared with wild-type kidneys.

### 3.2. Immune Cell Infiltration Is Reduced in the Kidney of DbpA-Deficient UUO Animals

**Figure 5:**
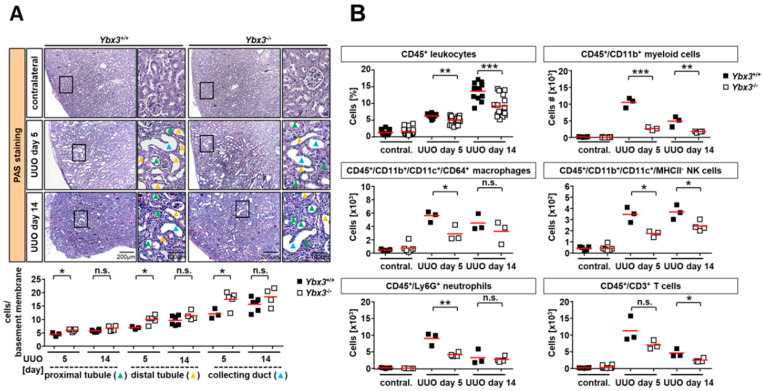
UUO-dependent tubular cell damage is not altered in *Ybx3*-deficient animals; however, immune cell infiltration is reduced. (**A**) Periodic acid–Schiff (PAS) staining of obstructed kidneys on days 5 and 14 showed no observable difference in the loss of brush border membranes or tubular dilation. In *Ybx3*-deficient mice, tubular cell death was determined by counting the number of cells per tubule. Scale bars represent 200 μm and 100 μm as indicated. Quantification is provided for wild-type and knockout animals. (**B**) Flow cytometry analyses of infiltrating immune cells on days 5 and 14 are shown for CD45+ leukocytes, myeloid cells (CD45+/CD11b+), macrophages (CD11b+/CD11c+/CD64+), NK cells (CD11b+/CD11c+/MHCII-), Ly6G+ neutrophils, and T cells (CD45+/CD3+) from both wild-type and knockout animals (* *p* < 0.05, ** *p* < 0.01, *** *p* < 0.001).

To assess tissue damage, we performed periodic acid–Schiff (PAS) staining on contralateral, 5-day, and 14-day UUO kidney sections from both wild-type and *Ybx3*-deficient mice (Figure 5A). Analysis of the 5-day and 14-day UUO sections from the wild-type mice showed the expected induction of tubular damage, as evidenced by the large vacuole-like structures resulting from cell death. *Ybx3*-deficient mice demonstrated a significantly higher number of cells attached to the basement membranes along the nephron, starting from the proximal to distal tubular segments and the collecting duct on day 5 of UUO. These differences were no longer seen on day 14 of the disease, suggesting that the loss of DbpA protects against the immediate early damage caused by UUO.

One component of tubulointerstitial nephritis is an increased recruitment of immune cells into the kidney following disease induction. In order to better quantify the number of immune cells present, the kidneys were harvested after 5 or 14 days of UUO and analyzed via flow cytometry following tissue disruption. Flow cytometry showed no difference in the number of leukocytes in the healthy contralateral kidneys of wild-type and *Ybx3* knockout animals. However, following the induction of UUO, we observed an increase in the number of infiltrating CD45-positive cells in both wild-type and *Ybx3* knockout animals; however, the number of infiltrating cells was reduced in *Ybx3* knockout mice compared to the wild type (Figure 5B and Figure S3). Among the CD45+ subsets, we observed a similar trend of reduced numbers of infiltrating CD11b+ myeloid cells, macrophages, NK cells, neutrophils, and CD3+ T cells. Importantly, no differences were observed in the blood, indicating that the lack of cell infiltration does not result from a hematopoietic defect in these animals (Figure 3 and Figure S4).

### 3.3. UUO-Induced Renal Interstitial Fibrosis Is Reduced in the Absence of Ybx3 In Vivo

**Figure 6:**
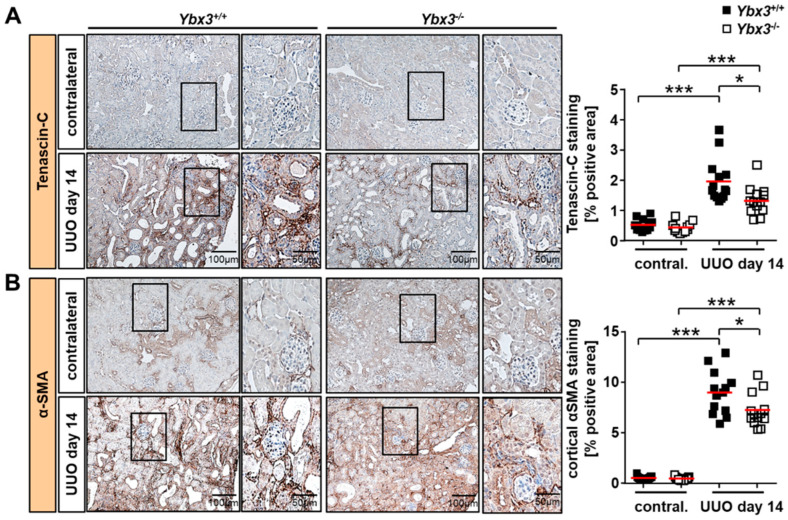
UUO-dependent renal tubulointerstitial fibrosis is reduced in *Ybx3*-deficient mice. Representative images of obstructed kidneys on day 14 in wild-type and *Ybx3* knockout mice stained for (**A**) tenascin-C and (**B**) α-smooth muscle actin (αSMA) are shown. Scale bars represent 100 μm and 50 µm. Quantification was performed by assessing the positively stained cortical area (%) in wild-type and knockout animals. Data obtained via computer-based morphometric analysis on day 14 after UUO induction in obstructed and ‘healthy’ contralateral kidneys showed decreased α-smooth muscle actin expression in fibrotic material in the *Ybx3* knockout compared with wild-type animals. Mean values are shown with red bars (* *p* < 0.05, *** *p* < 0.001).

Since maximum DbpA expression was observed late (day 14) and appeared most abundant in the cortical renal interstitium, we focused our analysis on the development of renal fibrosis, a key feature in the progression of chronic kidney disease. Tenascin-C is a major component of the fibrogenic niche, which is known to be upregulated in UUO [47]. Whereas wild-type mice showed the expected induction of tenascin-C expression upon disease induction, *Ybx3* knockout mice showed a marked reduction in tenascin-C expression compared to the wild type (Figure 6A).

Cortical alpha smooth muscle actin (αSMA) is considered a marker of myofibroblast activation and thus fibrosis. αSMA staining of healthy kidneys showed a predominant vascular pattern similar to that of DbpA. However, following disease induction, a marked increase in αSMA positivity was observed within the interstitium of WT mice, which was significantly reduced in the absence of DbpA (Figure 6B).

**Figure 7:**
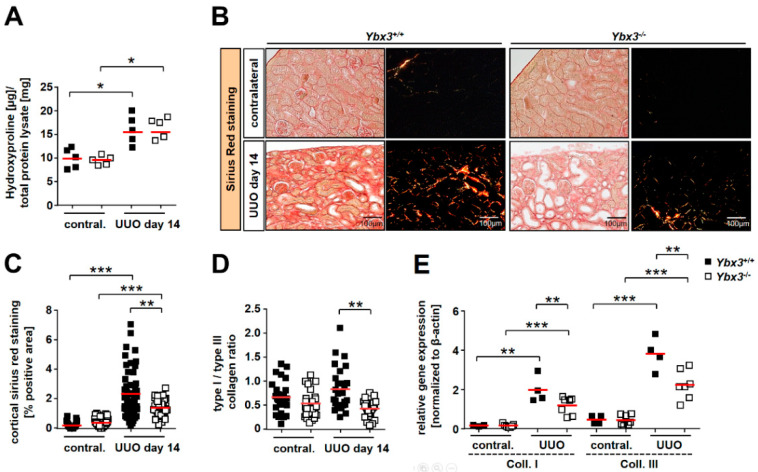
Collagen I and III synthesis is reduced in *Ybx3* knockout animals compared to wild-type animals. (**A**) Total collagen levels were estimated by determining the hydroxyproline content of the tissue for wild-type and knockout animals. (**B**) Sirius red staining of healthy and obstructed kidneys on day 14 from wild-type and *Ybx3* knockout mice (scale bars, 100 μm). (**C**) Quantification was performed by assessing the positively stained cortical area (%) for wild-type and knockout animals. The diagram shows data obtained via computer-based morphometric analysis on day 14 after UUO induction. Sirius red staining for collagen deposits showed less fibrotic material in knockout animals compared to the wild type. (**D**) Further analysis of the type I/type III collagen expression ratio showed a significant decrease in the type I/type III ratio in the *Ybx3* knockout group. (**E**) TaqMan analysis of the relative *Col1a1* and *Col1a3* transcripts in healthy contralateral and obstructed kidneys. The analysis showed a significant reduction in collagen transcripts in the absence of *Ybx3*. Data represent means ± SDs. Quantification is provided for wild-type and knockout animals (* *p* < 0.05, ** *p* < 0.01, *** *p* < 0.001).

To measure the changes in total collagen, tissue lysates were hydrolyzed, and the hydroxyproline content was determined. As expected, the collagen content increased upon disease induction, consistent with the development of fibrosis; however, no differences were observed between wild-type and knockout mice (Figure 7A). Sirius red staining of the tissue showed enhanced collagen deposition in wild-type mice after disease induction that was reduced in the *Ybx3* knockout (Figure 7B,C). Utilizing polarized light to determine the ratio of collagen type I to type III showed that this too was altered between the wild type and knockout (Figure 7D). The determination of transcript numbers via qRT-PCR confirmed the induction of both collagen type I and type III following UUO, which is consistent with the development of fibrosis. However, PCR analysis showed that transcripts for both collagen type I and type III were reduced in the *Ybx3* knockout animals compared with the wild type (Figure 7E).

## 4. Discussion

Here, we show, for the first time, that *Ybx3* deletion does not appear to influence kidney development or function in healthy mice. However, following ureter ligation, we observed a strong induction of the DbpA protein within the renal tubulointerstitium in wild-type mice. We observed a significant difference in tissue damage, especially tubular cell loss, on day 5 of disease, along with a significant reduction in the number of infiltrating immune cells in *Ybx3* knockout animals. Since we observed no differences within the composition of cells within the blood, this suggests that DbpA does not play a role in hematopoiesis. Considering that DbpA is expressed in the vasculature of healthy kidneys and that immune cell infiltration is reduced by the loss of DbpA, we cannot exclude a potential role for DbpA in regulating either the expression or activation of adhesion molecules (e.g., selectins and integrins) that are used by immune cells for transmigration.

However, given that the expression pattern of DbpA-positive cells in the tubulointerstitium is remarkably similar to that of CD11c+ tissue-resident renal dendritic cells (DCs), it may be that DbpA protein expression is induced in renal DCs upon activation in response to tissue injury. Renal DCs share a number of markers with infiltrating monocytes/macrophages (e.g., F4/80, CD11b, MHC-II), making it difficult to distinguish these cell types from one another. Renal DCs adopt a pro-inflammatory phenotype following ureter obstruction, even though they do not appear to directly contribute to fibrosis [48]. This is consistent with our previous observations that immune cell infiltration and fibrosis are distinct and separable events [29]. In this context, dendritic cells within the renal interstitium are ideally positioned for immune surveillance, as their foot processes extend into the tubules, allowing them to take up antigens [49]. Here, renal DCs play an important role in initiating immune cell infiltration by acting as sentinels and producing chemokines in response to damage-associated molecular patterns (DAMPs) released during tissue injury [50]. DCs also express receptors of the C-type lectin (CLEC) family, such as the mannose receptor, which bind collagen types I–IV via a fibronectin type II domain [51].

In the kidney, pericytes and fibroblasts are recognized as the primary sources of myofibroblasts, which are the major matrix-producing cells, including collagen type I [52]. These cells are clearly present in the tubulointerstitium, consistent with our data. Further investigation of the key markers of renal fibrosis showed a reduced induction of both tenascin-C and αSMA in *Ybx3* knockouts, as well as collagen I and III. The reduction in collagen expression may result from an increase in the YB-1 protein, as YB-1 is a known negative regulator of collagen expression [53]. Overall, the data clearly indicate a role for DbpA in supporting tissue fibrosis.

The hypothesis that *Ybx3* expression is induced in activated fibroblasts is supported by single-cell RNA sequencing data from the kidneys of UUO mice (Figure 4C) [42,43]. This dataset also reports DbpA expression in proliferating proximal tubules, which we did not observe. However, since DbpA is released from the cell membranes when tight junctions are disrupted, and translocates to the nucleus to drive proximal tubule cell proliferation, one cannot rule out the possibility that an experimental artifact was introduced into the dataset during the preparation of the tissue for single-cell analysis [21].

There are several possible mechanisms by which DbpA may regulate fibrosis. First, as a member of the cold shock domain protein family, DbpA is similar to YB-1 in both structure and function. Both proteins share an evolutionarily conserved cold shock domain that binds single-stranded RNA/DNA [4]. Recently, it was demonstrated that both proteins share an overlapping affinity for substrates, which is not surprising, as both proteins are found within the ribonucleoprotein complexes that regulate RNA stability and splicing [41]. Therefore, one possible mechanism is that DbpA translationally regulates the mRNA of tenascin-C and αSMA. However, since fibrosis is determined by the rate of extracellular matrix production as well as the rate of degradation, the latter being regulated by the levels of matrix metalloproteases (MMPs) and their inhibitors (TIMPs), it is foreseeable that DbpA could exert its influence by regulating the expression of these proteins as well. To further complicate matters, both microRNAs and lncRNAs are known to exert regulatory effects on mRNA expression and are themselves regulated by cold shock proteins [54,55]. Since the cold shock domain is conserved between Ybx1 and Ybx3, it is possible that some of the beneficial effects observed upon targeting YB-1 are indeed mitigated by DbpA. It is interesting to speculate that manipulating factors that are responsible for inducing DbpA expression may be a means to treat renal fibrosis.

## 5. Conclusions

Here, we report disease-specific upregulation of *YBX3* transcripts within different compartments of the kidney in patient biopsies, which agrees with our previous immune histochemical findings [7]. Since growth and kidney function do not appear to be influenced by the deletion of *Ybx3*, we can conclude that DbpA is not as essential for development as *Ybx1* has been shown to be [56,57]. Following disease onset, we showed a strong induction of the short (50 kDa) isoform of the Ybx3 protein (DbpA_b) in the kidney interstitium, where the DbpA-positive cells reside. How the two isoforms differ in function remains to be shown. However, the reduction in immune cell infiltration and the reduced expression of fibrotic markers in knockout mice lead us to conclude that DbpA upregulation contributes to the development of fibrosis in our disease model. Whether this conclusion holds up in other disease models and other organs has yet to be demonstrated. However, it is interesting to speculate that targeting DbpA may be a means to slow the development of fibrosis and thereby prolong kidney function.

## Data Availability

Not applicable.

## Supplementary Materials

The following supporting information can be downloaded at: https://www.mdpi.com/article/10.3390/cells12101426/s1, Figure S1: Gating strategy; Figure S2: Sequence homology; Figure S3: Flow cytometry analyses; Figure S4: Blood composition.
