# Supplementary material for: Cold Shock Domain Protein DbpA Orchestrates Tubular Cell Damage and Interstitial Fibrosis in Inflammatory Kidney Disease
Source: Cells. 2023 May 19;12(10):1426. doi: 10.3390/cells12101426 (PMC10217384; doi:10.3390/cells12101426)
Supplement: Supplementary file 1 [file cells-12-01426-s001.zip › cells-2373667-supplementary.pdf]

cells

MDPI

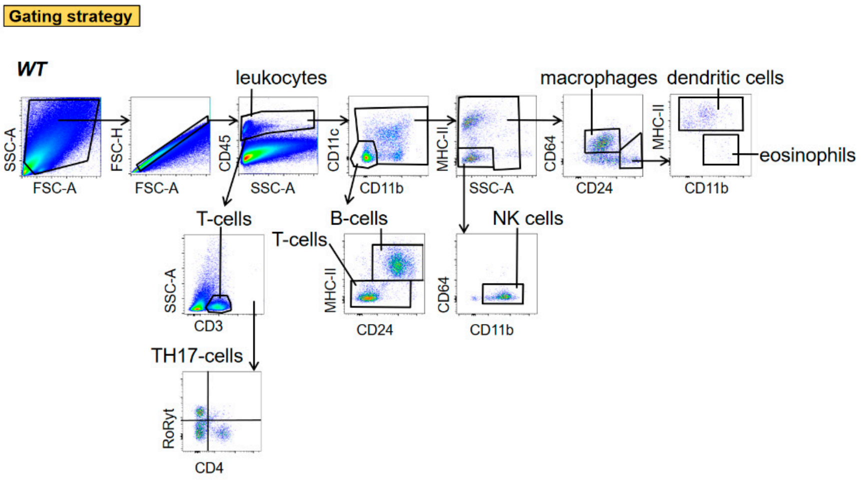

Supplementary Figure S1. Gating strategy for flow cytometric analysis of infiltrating immune cells.

A

CSDA_CRA_a [mouse]
CSDA_CRA_a [rat, EDM01693.1]

```
MESPREHSSRLIGIRLTARGSAGDLLAHDLPLLLERLKTKKTSKRLMVQTSRLPAVDSDV
:::::: ::: ::: ::: ::::::::::::::::::: :: ::: :::::::::::::
MESPRERSSRFIGIPLTAQGSAGDLLAHDLPLLLERLKIKKISKRPMVQTSRLPAVDSDA

PTTIGAVPVPSTLFHKMAKRPRQVKHQLRTPLQLPNRAVPSDPGSQAP _mouse
::: :: :::::::::::::::::::::::::: ::::::::::::::
PTTTGAAPVPSTLFHKMAKRPRQVKHQLRTPLQPPNRAVPSDPGSQAPSPPAG _rat
```

B

KO_b [mouse]

```
MSEAGEATTGGTTLPQAAADAPAAAPPDPAPKSPAASGAPQAPAPAALLAGSPGGDAAPG
PAPASSAPAGGEDAEKKVLGDLLAHDLPLLLERLKTKKTSKRLMVQTSRLPAVDSDVPTT
IGAVPVPSTLFHKMAKRPRQVKHQLRTPLQLPNRAVPSDPGSQAP
```

Supplementary Figure S2. (**A**) The sequence homology between the translated protein sequences for mouse and rat CRA_a is depicted. Colons indicate conserved amino acids. (**B**) The amino acid sequence of the translated KO_b fusion protein is presented.

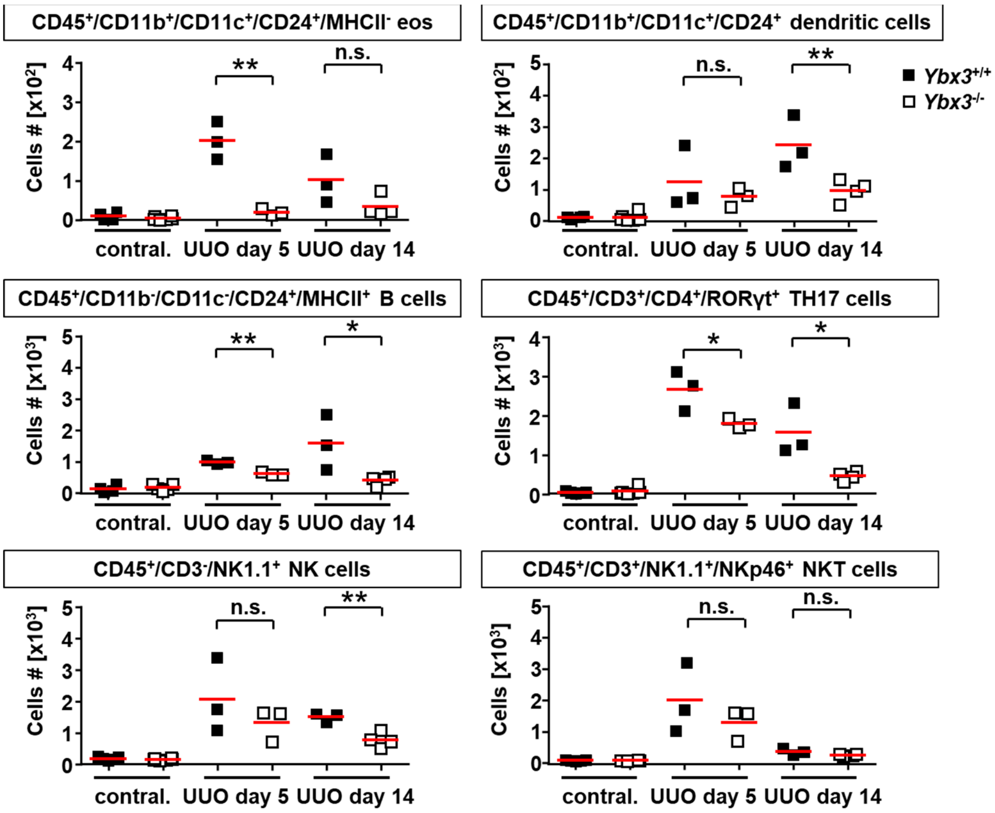

Supplementary Figure S3. Flow cytometry analyses of infiltrating immune cells at day 5 and 14 is presented for eosinophils (CD11b+/CD11c+/CD24+/MHCII-), dendritic cells (CD11b+/CD11c+/CD24+), B-cells (CD11b-/CD11c-/CD24+/MHCII+), TH17-cells (RORγt+/CD4+), NK cells (CD3-/NK1.1+), and NKT cells (CD3+/NK1.1+/NKp46+) from wild type and knockout animals.

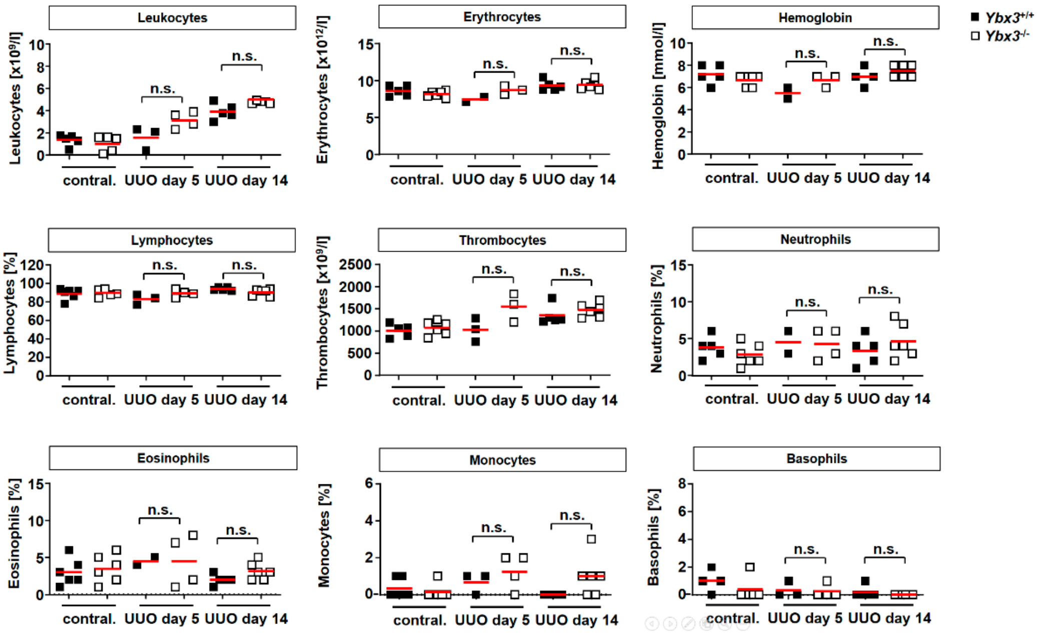

Supplementary Figure S4. Blood composition of wild type and knockout mice was analyzed after 5 and 14 days of UUO. Mean values are indicated by the red line. No significant differences in blood values were observed.
